# Work disability and employment status among advanced chronic kidney disease patients

**DOI:** 10.1371/journal.pone.0297378

**Published:** 2024-03-27

**Authors:** Shing Shen Bay, Lydia Kamaruzaman, Rozita Mohd, Shamsul Azhar Shah

**Affiliations:** 1 Department of Medicine, Hospital Canselor Tuanku Muhriz, Bandar Tun Razak, Kuala Lumpur, Malaysia; 2 Department of Medicine, Faculty of Medicine, Universiti Kebangsaan Malaysia, Kuala Lumpur, Malaysia; 3 Department of Community Health, Faculty of Medicine, Universiti Kebangsaan Malaysia, Kuala Lumpur, Malaysia; National Healthcare Group, SINGAPORE

## Abstract

**Introduction:**

Chronic kidney disease (CKD) is a major public health issue with significant socioeconomic impacts. In Malaysia, the prevalence of CKD in 2018 was 15%. Complications of CKD such as anaemia, mineral bone disease, and infections led to frequent hospitalizations resulting in work disability and unemployment. To date, there is no data of employment status of CKD patients in Malaysia.

**Methods:**

A cross-sectional study of patients with advanced CKD (stage 4 and 5 non-dialysis) treated in our centre. We interviewed those aged 18 to 60 years old who were selected based on random sampling of their employment status and associated factors. Work disabilities and quality of life were assessed using work productivity and activity impairment (WPAI-GH) questionnaire and kidney disease and quality of life (KDQOL-36) questionnaire. These questionnaires were assisted by the main investigators to aid participants in facilitating their response process.

**Result:**

A total of 318 patients recruited, 53.5% were males, with a mean age of 49.0 ± 9.0 years old. The main cause of CKD was diabetes (67.0%) followed by hypertension (11.3%). Majority of them were obese (55.3%) with a mean body mass index of 28.81 ± 6.3 kg/m^2^. The mean household income was RM 4669.50 ± 3034.75 (USD1006.27 ± 653.99). The employment rate was 50% (n = 159). 86% of the unemployed patients were in B40 income category. Multiple Logistic Regression was performed on the significant factors affecting employment status showed one year increase in age increased 6.5% odds to be unemployed. Female and dyslipidaemia had 2.24- and 2.58-times higher odds respectively to be unemployed. Meanwhile, patients with tertiary level of education were 81% less odds to be unemployed. Patients with advanced CKD had a mean percentage of 24.35 ± 15.23 work impairment and 13.36 ± 32.34 mean percentages of face absenteeism due to the disease burden. Furthermore, patients who were unemployed had significant perceived symptoms and problem lists, effects, and burden of kidney disease (p<0.01) and showed poor mental and physical composites (p<0.01) as compared with those who were employed.

**Conclusion:**

The employment rate of advanced CKD patients was low with half of patients lost their jobs due to the disease burden and had poor mental and physical composites of quality of life. This raises the concern for financial support for long term renal replacement therapy.

## 1.0 Introduction

Chronic kidney disease (CKD) is a significant public health issue associated with high morbidity and mortality [1]. Its impact on social and economic aspects is particularly pronounced in low and middle-income countries [2]. The global prevalence of CKD was reported to be around 11%-13% in 2016 [3]. A population-based study in 2018 found that 15% of Malaysians had CKD, with 0.25% and 0.08% of them classified as being in CKD stages 4 and 5, respectively [4]. CKD is associated with multiple complications such as loss of kidney function leading to end-stage renal disease (ESRD), anaemia, CKD-mineral bone disease, and infections [5]. Due to low transplant rates, advanced age, and underlying comorbidities, most ESRD patients remain on dialysis.

Advanced CKD includes stages 4 and 5, characterized by a severe reduction in glomerular filtration rate (GFR) to < 30ml/min [6, 7]. During this stage, patients may experience symptoms that interfere with their daily lives, impacting their quality of life and employment rates [5]. Symptoms commonly include fatigue, weakness, decreased mental sharpness, shortness of breath, and more [8]. Fatigue may be attributed to anaemia, as lower haemoglobin levels have been shown to be associated with greater loss of work productivity and activity impairments, leading to increased economic consequences [9]. Anxiety and depression are also prevalent among CKD patients in Malaysia, with almost 11% of patients experiencing these conditions, likely resulting from reduced physical functioning caused by fatigue, body pain, and daily life restrictions [10].

Education attainment is another factor influencing employment rates in the CKD population, favouring highly educated individuals [11]. In China, individuals with higher education are more likely to find and retain employment compared to those with lower education [12]. Age and gender have also been recognized as factors affecting employment rates. Younger workers are generally preferred over older workers due to perceived productivity advantages [13]. Male workers tend to have higher employment rates compared to females, as female workers often experience labour market disruptions due to family formation and childcare responsibilities [14].

In Malaysia, the cost of haemodialysis treatment is approximately RM39,790 (USD8754) per annum, while peritoneal dialysis costs around RM37,576 (USD8267) per annum [15]. Based on the Malaysian Dialysis and Transplant Registry (MDTR) data 2021, the government remains the primary funding source for dialysis, supporting 43% of new dialysis patients and 48% of existing dialysis patients [16]. Approximately a quarter of new dialysis patients are self-funded, while 16% and 9% receive funding from Employees’ Social Security Organization (SOCSO) as well as contributions from charitable sources for social welfare, respectively. Medical insurance is not a significant contributor, as less than 1% of ESRD patients are funded through insurance.

Insurance coverage is inadequate among Malaysians with CKD. Having insurance coverage can benefit CKD patients, as demonstrated by the Kidney Early Evaluation Program in 2013, which found that 72% of uninsured patients were likely to progress to ESRD compared to insured patients [17]. CKD imposes a heavy financial burden on the healthcare system in Malaysia, with approximately 23% of stage 5 CKD patients requiring financial aid from the public sector in 2019 [18].

In Malaysia, household income is categorized into three groups: B40 represents the bottom 40% of earners, M40 represents the middle 40%, and T20 represents the top 20% [19]. The B40 group has a household income of <RM4,850 (USD1067) per month, the M40 group falls between RM4,851 (USD1067) and RM10,960 (USD2411) per month, while the T20 group earns >RM10,961 (USD2411) per month [19]. Lower-income Malaysians face socioeconomic challenges, health disparities, and food insecurity. Their low-income status hampers access to healthcare and a conducive living environment, resulting in poorer quality of life [20].

The primary aim of this research was to ascertain the prevalence of work disability and employment rates among individuals with advanced chronic kidney disease (CKD). Furthermore, this study aimed to uncover potential factors linked to work disability and employment status in advanced CKD patients, while also evaluating the overall quality of life of these patients. Currently, there is no available statistics regarding the employment rate for patients with advanced CKD in Malaysia. Considering the mentioned risk factors and retrieved details, it would be beneficial to analyse the factors influencing employment, work impairments, and quality of life among patients with advanced CKD.

### 1.1 Study hypothesis

1. Patients with advanced CKD has higher work disability rate and low employment rate compared to the general population
2. Renal anemia, fluid overload, depression and anxiety are associated with lower employment rate
3. Patient with advanced CKD has low quality of life

## 2.0 Methodology

### 2.1 Study design

This study was a cross-sectional approach, utilizing interview-based questionnaires, to investigate individuals with advanced chronic kidney disease (CKD) falling within the age range of 18 to 60 years. The interview-questionnaire method was used to aid participants, facilitating their response process. Participants were recruited based on random sampling from those attending follow-up appointments at the nephrology clinic or those admitted to the medical wards at Hospital Canselor Tuanku Muhriz, Universiti Kebangsaan Malaysia, during the period spanning October 1, 2021, to May 31, 2023. The selection of participants was conducted using simple random sampling method. All participants provided their consent prior to interview either verbally or in writing.

### 2.2 Ethics considerations

This study has been approved by the ethical board of the National University of Malaysia (FF-2021-397) in accordance with the International Conference of Harmonization Good Clinical Practice Guidelines.

### 2.3 Sample size

As Malaysia has not have any data on employment rate in patients with advanced CKD, hence sample size calculation is based on employment rate in Netherland 2021, which showed prevalence of employment rate of 68%. The sample size was calculated based on the formula below with the confidence level of 90% and maximum error permissible of 0.05.


$$\text{n}=\frac{\left(z^{2}\right)P\left(1-P\right)}{d^{2}}$$

Z = degree of confidence (90%)

P = expected prevalence: 0.68

d = max error: 0.05

The sample size of this study is 237 patients. However, we were able to recruit a total of 318 participants for this study in the end.

### 2.4 Study tools

Two standardised validated questionnaires either in English or Malay were used; 1) work productivity and activity impairment (WPAI-GH) questionnaire and 2) kidney disease and quality of life (KDQOL-36) questionnaire.

#### 2.4.1 WPAI:GH questionnaire

Work Productivity and Activity Impairment—General Health (WPAI:GH) questionnaire was created as a patient-reported quantitative assessment [7, 21]. This questionnaire has 6 questions, which are used to calculate 4 types of scores; which are absenteeism (work time missed), presenteeism (reduced on the job effectiveness), work productivity loss (overall work impairment) and activity impairment. The scores are expressed in percentage with higher numbers indicating greater impairment and less productivity [22, 23].

#### 2.4.2 KDQOL-36 questionnaire

Kidney Disease Quality of Life (KDQOL-36^™^) has 36 questions for assessment of health-related quality of life among dialysis patients. This tool contains 5 subscales: The Physical Component Summary (PCS), Mental Component Summary (MCS), Burden of Kidney Disease (BKD), Symptoms and Problems of Kidney Disease (SPKD) and Effect of Kidney Disease (EKD). The first 2 subscales are generic measures of Health-Related Quality of Life (HRQOL) [12 items in total] whereas the last 3 subscales assess issues specific to patients with ESRD or CKD [24 items in total]. Each of the KDQOL-36 kidney-targeted scales are scored by transforming all items linearly to 0–100 possible range and averaging the items in the scale. High scores indicate better health related quality of life [24, 25].

## 3.0 Stastistic analysis

Data were analysed using SPSS software, version 25.0 (SPSS Inc. Chicago, IL). Data was expressed as mean ± standard deviation (SD) as all data were normally distributed. Independent t-test was used for comparison between numerical variables while Chi Square was used to compare two categorical data. Logistic Regressions were used to determine factors associated with unemployment and work disability among dialysis patients. The p ≤ 0.05 is considered significant.

## 4.0 Results

### 4.1 Socio-demographic and prevalence

A total of 318 patients were recruited with the mean age of 49.13 ± 9.422 years old and 53.5% were males. The majority (74.8%) were Malay. Most of our patients were obese (55.3%) based on the Asian BMI classification. Diabetes mellitus was as the main cause of ESRD (67%). Half of the populations (50%) were employed (Table 1).

**Table 1:** Baseline socio-demographic data of study population.

Parameters	(N = 318, %)
Age (years)	49.13 ± 9.422
Gender	
Male	170 (53.5)
Female	148 (46.5)
Ethnicity	
Malay	238 (74.8)
Chinese	51 (16.0)
Indian	23 (7.2)
Others	6 (1.9)
Body mass index, n (%)	
Underweight <18.5 kg/m^2^	14 (4.4)
Normal 18.5–22.99 kg/m^2^	47 (14.8)
Overweight 23.0–27.5 kg/m^2^	81 (25.5)
Obese ≥ 27.5 kg/m^2^	176 (55.3)
Primary Renal Disease	
Diabetes Mellitus	213 (67.0)
Hypertension	36 (11.3)
Glomerulonephritis	28 (8.8)
Obstructive Uropathy	14 (4.4)
Analgesic Nephropathy	2 (0.6)
Polycystic Kidney Disease	3 (0.9)
Congenital and Hereditary	3 (0.9)
Autoimmune	7 (2.2)
Unknown	12 (3.8)
Comorbidities	
Hypertension	295 (92.8)
Diabetes mellitus	227 (71.4)
Dyslipidaemia	279 (87.7)
Ischemic heart disease	68 (21.4)
Marital status	
Married	262 (82.4)
Single	37 (11.6)
Divorced/Separated/Widow	19 (6.0)
Education level	
Primary level	13 (4.1)
Secondary level	170 (56.3)
Tertiary level	126 (39.6)
Employment status	
Employed	159 (50.0)
Unemployed	159 (50.0)
Type of employment	
Full time	140 (46.9)
Part time	6 (1.9)
Self employed	4 (1.3)
Employment sector	
Private	169 (53.1)
Government	93 (29.2)
Income (Ringgit Malaysia)	2193.87 ± 2459.19
Household income	4669.50 ± 3034.75
Income category	
B40	174 (54.7)
M40	125 (39.3)
T20	19 (6.0)

Continuous values are presented as mean ± standard deviation for normally distributed data and median (25^th^ percentile; 75^th^ percentile) for non-normally distributed data.

Table 2 illustrated the associated factors for employment status among the advance CKD patients. Patients who were unemployed were significantly older (p < 0.001) with the mean age of 52.20 ± 8.28 years old. Predominantly female patients were unemployed (p = 0.01). Patients who had diabetes mellitus (p = 0.003) and dyslipidaemia (p < 0.001) tended to be unemployed too. Those who were unemployed mostly consisted of or contributed to the B40 group (p < 0.001). On the other hand, patients who had tertiary educations were likely to be employed (p < 0.001).

**Table 2:** Associated factors for employment status of patients with advanced CKD.

Parameters	Employed (n = 159)	Unemployed (n = 159)	p-value
Age (years)	46.1 ± 9.5	52.2 ± 8.3	**<0.001** _a_
Gender, n (%)			**0.010** _b_
Male	97 (61.0)	73 (45.9)	
Female	62 (39.0)	86 (54.1)	
Race, n (%)			0.386_b_
Malay	116 (73.0)	122 (76.7)	
Chinese	27 (17.0)	24 (15.1)	
Indian	11 (6.9)	12 (7.6)	
Others	5 (3.1)	1 (0.6)	
Body mass index, n (%)			0.095_b_
Underweight	5 (3.1)	9 (5.7)	
Normal	25 (15.7)	22 (13.8)	
Overweight	49 (30.8)	32 (20.1)	
Obese	80 (50.3)	96 (60.4)	
Comorbidity, n (%)			
Hypertension	145 (91.2)	150 (94.3)	0.387_b_
Diabetes mellitus	101 (63.5)	126 (79.3)	**0.003** _b_
Dyslipidaemia	129 (81.1)	150 (94.3)	**<0.001** _b_
Ischemic heart disease	31 (19.5)	37 (23.3)	0.494_b_
Marital status, n (%)			0.203_b_
Married	136 (85.5)	126 (79.2)	
Single	17 (10.7)	20 (12.6)	
Divorced/separated/widow	6 (3.8)	13 (8.2)	
Highest education level, n (%)			**<0.001** _b_
Primary level	6 (3.7)	7 (4.4)	
Secondary level	64 (40.3)	115 (72.3)	
Tertiary level	89 (56.0)	37 (23.3)	
Income category, n (%)			**<0.001** _b_
B40	37 (23.3)	137 (86.2)	
M40	103 (64.8)	22 (13.8)	
T20	19 (11.9)	0 (0)	
Anaemia, n (%)	54 (33.96)	70 (44.03)	0.085_b_
Haemoglobin (g/dL)	11.1 ± 2.3	10.3 ± 1.8	**0.001** _a_

_a_ independent t-test,

_b_ Pearson Chi Square test

Continuous values are presented as mean ± standard deviation for normally distributed data and median (25^th^ percentile; 75^th^ percentile) for non-normally distributed data.

### 4.2 Logistic regression

Both Simple and Multiple Logistic Regression Analysis were performed to identify the significant factors that affecting employment status as shown in Table 3. A cut-off points of 0.25 was set for variable selection in Simple Logistic Regression to be included in Multiple Logistic Regression [26, 27] as individual variables that are weakly correlated with the outcome might contribute significantly when combined in multivariable analysis. The traditional cut-point of 0.05 may result in important variables being removed from the model [28, 29].

**Table 3:** Logistic regression of the significant associated factors affecting employment status.

Variables	Simple Logistic Regression		Multiple Logistic Regression	
	Crude Odds Ratio (95% CI)	p-value	Adjusted Odds Ratio (95%CI)	p-value
Age (years)	1.08(1.05,1.11)	**<0.001**	1.07(1.04,1.10)	**<0.001**
Gender				
Male	1		1	
Female	1.84(1.18,2.88)	**0.007**	2.24(1.29,3.88)	**0.004**
Race				
Malay	1		1	
Chinese	0.85(0.46,1.55)	0.586	0.72(0.36,1.45)	0.360
Indian	1.04(0.44,2.44)	0.933	0.89(0.34,2.34)	0.810
Others	0.19(0.02,1.65)	0.132	0.13(0.01,1.73)	0.121
Body mass index				
Normal	1			
Underweight	2.05(0.60,7.03)	0.256		
Overweight	0.74(0.36,1.53)	0.421		
Obese	1.36(0.72,2.60)	0.346		
Hypertension				
No	1			
Yes	1.61(0.68,3.83)	0.283		
Diabetes mellitus				
No	1		1	
Yes	2.19(1.33,3.62)	**0.002**	1.11(0.58,2.12)	0.746
Dyslipidaemia				
No	1		1	
Yes	3.88(1.78,8.47)	**0.001**	2.58(1.01,6.59)	**0.048**
Ischemic heart disease				
No	1			
Yes	1.25(0.73,2.14)			
Marital status				
Single	1			
Married	0.79(0.40,1.57)	0.498		
Divorced/Others	1.84(0.58,5.90)	0.304		
Highest education level				
Primary level	1		1	
Secondary level	1.54(0.50,4.78)	0.455	0.83(0.19,3.71)	0.805
Tertiary level	0.36(0.11,1.13)	**0.080**	0.19(0.04,0.87)	**0.032**
Haemoglobin	0.84(0.75,0.93)	**0.002**	0.84(0.69,1.03)	0.088
Anaemia				
No	1		1	
Yes	1.53(0.97,2.41)	**0.066**	0.98(0.43,2.25)	0.965

There were eight variables (age, gender, race, diabetes mellitus, dyslipidaemia, highest education level, haemoglobin, and anaemia) found to be potentially significant (p<0.25) hence included in Multiple Logistic Regression Analysis. The Multiple Logistic result showed that model explained 33.1% (Nagelkerke R^2^) of the variance in employment status and correctly classified 70.8% of cases. The Hosmer Lameshow demonstrated non-significant result (p = 0.615), which indicate that model adequately fits the data and the predicted results were reliable. 4 variables were found to be significant (p<0.05). These were age, gender, dyslipidaemia, and highest education level.

1. One-year increase in age increased 6.5% odds (chance) to be unemployed (p <0.001)
2. Female was 2.24 times higher odds (chance) to be unemployed compared to male (p = 0.004)
3. Dyslipidaemia was 2.58 times higher odds (chance) to be unemployed (p = 0.048)
4. Patients with tertiary level of education was 81% less odds (chance) to be unemployed compared to patients with primary level of education (p = 0.032)

### 4.3 Work productivity and activity impairment

Table 4 showed those who were employed had a mean percentage of 13.36% ± 32.34 missed their work due to the health issue, while 24.35% ± 15.23 percent of them had overall work impairment due to their health.

**Table 4:** Work productivity and activity impairment of employed patients with advanced chronic kidney disease.

Parameters	N = 159
Percentages of work time missed due to health	13.36 ± 32.34
Percentages of impairment while working due to health	51.32 ± 15.23
Percentages of overall work impairment due to health	24.35 ± 15.23

Continuous values are presented as mean ± standard deviation for normally distributed data and median (25^th^ percentile; 75^th^ percentile) for non-normally distributed data.

### 4.4 Quality of life assessment

Table 5 summarized the KDQOL-36 scores of our cohort. Those who were unemployed had a poorer quality of life due to kidney disease symptoms and effects, CKD burden and significantly lower both physical and mental composites scores (p < 0.001).

**Table 5:** KDQOL-36 domain subscale with employment status.

Variables	Employed (n = 159)	Unemployed (n = 159)	p-value
Symptoms/Problem list	82.03 ± 15.10	68.33 ± 18.63	**<0.001** _a_
(Mean ± SD)			
Effects of kidney disease	66.00 ± 19.92	43.42 ± 22.57	**<0.001** _a_
(Mean ± SD)			
Burden of kidney disease	69.26 ± 22.94	47.56 ± 28.70	**<0.001** _a_
(Mean ± SD)			
SF-12 Physical composite	46.04 ± 10.43	34.25 ± 10.36	**<0.001** _a_
(Mean ± SD)			
SF-12 Mental composite	48.59 ± 9.81	38.94 ± 11.05	**<0.001** _a_
(Mean ± SD)			

_a_ independent t-test

## 5.0 Discussion

Patients with advanced CKD face multiple challenges in employment due to their medical, psychological, and social circumstances. Our study investigated the employment rate in advanced CKD patients, revealing that only half of the population (50%) were employed. This finding was surprisingly lower compared to other countries such as the Netherlands, which reported employment rates of 68% in 2019, and India, which reported 61.1% in 2017 in patients with advanced CKD [30, 31]. The primary cause of advanced CKD in our study cohort was diabetes mellitus, which aligns with Hooi et al. in 2011, where diabetes mellitus was the top contributor to the primary cause of CKD [32]. Additionally, more than half of the patients (55.3%) were classified as obese (≥28 kg/m^2^) based on the Asian BMI classification [33], which was consistent with the 2015 National Health and Morbidity Survey (NHMS) that reported 51.2% of Malaysians being overweight/obese [34].

The mean age of unemployed patients with advanced CKD was 52.2 ± 8.3 years, which consistent with Alma et al., who reported that CKD patients aged over 54 years were less likely to be employed [31]. Female patients had a 2.24 times higher likelihood of being unemployed compared to males, as observed in our study. We hypothesized that this could be attributed to female patients being more involved in domestic tasks and child-rearing responsibilities, potentially choosing to be homemakers rather than pursuing a career. In 2015, 42.4% of Malaysian females reported stopping work, with 32.4% attributing it to household issues [35]. However, further investigation is warranted to understand the reasons behind being a housewife, as some females may prefer to be housewives to take care of their families, while others may be genuinely unemployed and choose to be housewives temporarily while waiting to be employed.

Patients with tertiary education levels (81%) were more likely to be employed, indicating that education level is a strong independent predictor for employment among CKD patients. We speculated that higher education levels may lead to job roles that require less physical labor and offer flexible working hours, unlike lower educational groups. Similar findings were reported by Curtin et al., who observed that patients with education and/or white-collar jobs were more likely to be employed [11].

Anemia and lower hemoglobin levels showed significant associations in the univariate analysis. In the study by Van Haalen et al., lower hemoglobin levels and anemia were highly associated with higher work disability and poorer health-related quality of life [36]. Despite hemoglobin being an associated factor (P = 0.001), unfortunately in our study, both hemoglobin and anemia did not show significance in the multivariate analysis. Thus, further studies with larger sample sizes should be conducted to explore these factors as potential contributors to unemployment in patients with advanced CKD.

No significant associations were found between employment rate in patients with advanced CKD and factors such as ethnicity, and body mass index. As Malaysia is a multicultural country, ethnicity is rarely a factor influencing employment. Similarly, Alma et al. reported no association between ethnicity and employment rate in CKD patients [31]. The lack of significant associations for factors such as body mass index may be due to comparable socio-demographic data between the employed and unemployed groups.

CKD can have varying degrees of impact on an individual’s ability to work, depending on disease severity and the presence of other complications. We found that employed patients with advanced CKD exhibited a mean percentage of 24.35 ± 15.23 overall work impairment due to health, along with a mean percentage of 13.36 ± 32.34 on absenteeism due to health issues. These results were similar to Van Haalen et al., who reported a mean percentage of 33 for overall work impairment and a mean percentage of 10 for absenteeism in patients with advanced CKD [36].

The quality of life of advanced CKD patients is often overlooked but is a critical consideration when evaluating overall medical care [34]. The results showed that unemployed patients had poorer quality of life compared to those who were employed. Their symptoms, disease burden, and mental and physical health were significantly impacted, hindering their employment prospects. Conversely, patients who managed to sustain employment were significantly less impacted by their kidney disease, resulting in a better health-related quality of life.

## 6.0 Strength of study

The data collected is represent the local population and portray the employment rate and factors affecting employment accurately around this population.

## 7.0 Limitation of study

The study limited generalizability arises due to its exclusive concentration on a single center’s population, potentially lacking representation of diverse demographic variations across various geographical regions. Besides that, the restriction of data collection to a single time point presents obstacles in establishing causation and understanding the direction of relationships. This limitation curtails insights into longitudinal patterns and the establishment of direct causal connections. On a side note, future research endeavors should consider analyzing depression and other psychological factors as potential influencers of work impairment and employment status among dialysis patients. Additionally, to comprehensively examine engagement in paid employment beyond household duties, further investigations should be directed towards the demographic of housewives.

## 8.0 Conclusion

The prevalence of employment among advance CKD patients is low due to disease burden causing them to have more work and activity impairment.
